# Signs Observed Among Animal Species Infected with Raccoon Rabies Variant Virus, Massachusetts, USA, 1992–2010

**DOI:** 10.3390/ani1040396

**Published:** 2011-11-18

**Authors:** Xingtai Wang, Barbara G. Werner, Sandra Smole, Vasil Pani, Linda L. Han

**Affiliations:** Bureau of Laboratory Science, Massachusetts Department of Public Health, 305 South Street, Jamaica Plain, Boston, MA 02130, USA; E-Mails: barbara.werner@state.ma.us (B.G.W.); sandra.smole@state.ma.us (S.S.); vasil.pani@state.ma.us (V.P.)

**Keywords:** raccoon rabies variant virus (RRV), clinical signs, rabid animals, aggression

## Abstract

**Simple Summary:**

We analyzed signs occurring among domestic and wild terrestrial animal species with raccoon rabies variant virus in Massachusetts, 1992–2010. While aggression is a useful predictor of rabies among wild animals, combinations of other signs such as ataxia, disorientation, and salivation are useful predictors of rabies among domestic animals.

**Abstract:**

We analyzed signs occurring among domestic and wild terrestrial animal species infected with raccoon rabies variant virus (RRV) in Massachusetts, 1992–2010. The clinical sign of aggression was significantly associated with rabid stray cats (odds ratio, OR = 2.3) and RRV affected major wild terrestrial animal species individually, which included raccoons (OR = 2.8), skunks (OR = 8.0), gray foxes (OR = 21.3), red foxes (OR = 10.4), woodchucks (OR = 4.7) and coyotes (OR = 27.6). While aggression is a useful predictor of rabies among wild animals, combinations of other signs such as ataxia, disorientation, and salivation are useful predictors of rabies among domestic animals. Pets reported with multiple clinical signs had significantly higher rabies positive testing result than those reported with single clinical sign (p < 0.001). The result suggested the importance of avoiding aggressive terrestrial wild animals and giving additional attention to pets with multiple clinical signs.

## 1. Introduction

The introduction of raccoon rabies variant (RRV) in Massachusetts in September 1992, resulted in an enormous and sustained increase in rabies cases among terrestrial animals [1]. While this dramatic shift in the incidence and epidemiology of animal rabies in MA has been well-characterized, questions remain regarding clinical features among individual animal species infected with the new virus variant. Understanding the behaviors of individual rabid animal species may be useful in assessing the risk of disease in symptomatic animals, particularly domestic animals.

In this study, we examine 19 years of rabies surveillance data to describe the frequency of various reported presenting signs among affected animal species.

## 2. Experimental Section

Specimens from animals suspected to have rabies are routinely submitted to the Massachusetts Department of Public Health William A. Hinton State Laboratory Institute (SLI) for laboratory testing. Specimens are submitted with a standard submission form that collects information on associated signs, including aggression, ataxia, disorientation, found dead, lethargy, paralysis, salivation and seizure. Specimens are tested for rabies with a standard direct fluorescent antibody test (DFA) in accordance with recommendations by the Centers for Disease Control and Prevention (CDC) [2]. All suitable non-raccoon terrestrial animals and a subset of positive raccoons were strain typed initially with a CDC panel of monoclonal antibodies and after 1999, with the Chemicon monoclonal antibody kit (Chemicon International, Temecula, CA, USA). All rabies cases among terrestrial animals were considered to be due to RRV, unless typing data revealed otherwise.

Reported signs were analyzed only for the nine predominant animal species submitted between 1992 and 2010 inclusive. Wild animals included raccoon (*Procyon lotor*), skunk (*Mephitis mephitis*), gray fox (*Urocyon cinereoargenteus*), red fox (*Vulpes vulpes*), woodchuck (*Marmota monax*) and coyote (*Canis latrans*). Domestic animals included cat (*Felis silvestris catus*), dog (*Canis lupus familiaris*) and cow (*Bos primigenius*).

All statistical analyses were performed with the SAS statistical package, version 9.1 (SAS Institute Inc., Cary, NC, USA). The chi-square test was used to compare differences in frequencies, with significance determined at α = 0.05 level. The association of each reported presenting sign with the positive rabies testing result was measured by the Odd Ratio (OR) and its 95% Confidence of Interval (95% CI).

## 3. Results and Discussion

A total of 54,919 testable specimens from terrestrial animal species were submitted to SLI for rabies diagnostic testing between 1992 and 2010 inclusive, including 40,148 (73.1%) specimens from the nine predominant terrestrial animal species. Among these 40,148 specimens, 4,461 (11.1%) were confirmed as having rabies infection. All were attributed to RRV infections, except for one imported dog infected with a mongoose strain of rabies virus (personal communication, CDC). The number of each species tested and the number attributed to RRV infection appear in Table 1.

Report of aggression was significantly associated with a positive rabies test result for all wild animal species examined (p < 0.0001) (Table 1). Among aggressive wild animals, the likelihood of rabies was greatest in coyotes (OR = 29.1, 95% CI = 6.17 − 137.37), followed by gray foxes (OR = 21.34, 95% CI = 12.12 − 37.56), red foxes (OR = 9.87, 95% CI = 5.27 − 18.51), skunks (OR = 7.99, 95% CI = 6.65 − 9.59), woodchucks (OR = 4.56, 95% CI = 2.96 − 7.03) and raccoons (OR = 2.88, 95% CI = 2.48 − 3.44). Six (60%) of 10 rabid coyotes had reported aggression, compared to five (4.9%) of 102 non-rabid coyotes (p < 0.0001). Similarly, 79 (74.53%) of 106 rabid grey fox exhibited aggression, compared to 34 (12.06%) of 282 non-rabid grey fox (p < 0.0001). Among 1,520 woodchucks tested, rabies was also associated with disorientation (OR = 3.9, 95% CI = 2.37 − 6.44), lethargy (OR = 4.39, 95% CI = 2.43 − 7.96) and paralysis (OR = 5.16, 95% CI = 1.84 − 14.52). Among 4,045 skunks tested, rabies was also associated with disorientation (OR = 1.95, 95% CI = 1.64 − 2.34). Among 5,081 raccoons tested, rabies was also associated with the animals being found dead (OR = 1.21, 95% CI = 1.0 − 1.46). There was no positive association between rabies and reports of ataxia, salivation, or seizure in any of the predominant wild animal species.

Among domestic animals, cats were the only species for which reported aggression was significantly associated with a positive rabies virus test result (OR = 1.53, 95% CI = 1.13 − 2.07) (Table 1). However, this association appears to be driven by non-pet cats (namely, wild, stray and cats of undefined sources) (OR = 2.1, 95% CI = 1.45 − 3.06). When pet cats only were considered, aggression was not associated with an increased likelihood of rabies (OR = 0.86, 95% CI = 0.5 − 1.48). Among 20,681 cats tested, rabies was associated with signs other than aggression, including paralysis (OR = 4.93, 95% CI = 3.4 − 7.15), ataxia (OR = 4.92, 95% CI = 3.63 − 4.69), disorientation (OR = 3.62, 95% CI = 2.67 − 4.92) and lethargy (OR = 2.36, 95% CI = 1.7 − 3.28). There was a borderline association between rabies and salivation (OR = 1.57, 95% CI = 0.98 − 2.51).

Among 7,782 dogs tested, aggression was not associated with RRV infection, with 5 (55.56%) of 9 RRV infected dogs exhibiting aggression, compared to 4,482 (57.67%) of 7,772 non-rabid dogs. However, rabies was strongly associated with salivation (OR = 19.08, 95% CI = 5.5 − 66.19), ataxia (OR = 14.4, 95% CI = 3.85 − 53.83), lethargy (OR = 7.84, 95% CI = 2.02 − 30.42) and disorientation (OR = 5.68, 95% CI = 1.47 − 22.04). Among 104 cows tested, only salivation (OR = 3.27, 95% CI = 1.06 − 10.07) was associated with rabies; 9 (60%) of 15 rabid cows had reported salivation, versus 28 (31.46%) of 89 non-rabid cows. Among 5,215 cats, dogs, and cows with more than one reported sign, 111 (2.08%) had rabies, compared to 88 (0.38%) of 23,151 with one or no reported signs (OR = 5.7, 95% CI = 4.3 − 7.55).

In general, having any of several reported associated signs was a better predictor of rabies than aggression or any other individual sign, in terms of positive predictive value (PPV), negative predictive value (NPV) and accuracy. More importantly, the relevant signs were different for wild and domestic animal species, with aggression being an important sign in rabid wild animal species, while combinations of other signs were more useful in predicting rabies in domestic animals (Table 2). Interestingly, the single sign of salivation had 24.32% PPV and almost 91.04% NPV in predicting RRV infection in cows.

We acknowledge several limitations of the study. The data in the rabies specimen submission forms are collected from a variety of individuals with a range of veterinary clinical assessment ability. Lay persons may be more likely to interpret as aggressive the behaviors of wild animals than those of domestic animals. This study is a retrospective review of data that were collected along with routine diagnostic specimens, without the benefit of active real-time data quality management and control. In addition, similar signs may be caused by other diseases, such as distemper, and in fact, only the laboratory examination of brain tissue by DFA can definitively diagnose rabies.

## 4. Conclusions

The association between animal aggression and rabies virus infection has been described for raccoon rabies variant as well as other rabies strains [3-5]. We had previously reported that signs of aggression, ataxia, disorientation and paralysis were significantly associated with RRV rabies, adjusting for wild versus domestic animals, animal species, and introduction of RRV in Massachusetts [6-8]. In this study, we demonstrate that report of aggression is associated with RRV rabies in wild animals, but may be less useful as a predictor of rabies in domestic animals. In domestic animals, the occurrence of multiple signs other than aggression is an important consideration in assessing the risk of rabies infection.

## Figures and Tables

**Table 1:** Reported clinical signs and likelihood of infection by raccoon rabies variant virus, by animal species, Massachusetts, 1992–2010 ^§^.

Reported Signs OR/p value	Animal species (No. of rabid/non-rabid animals)								

	Raccoon 2,434/2,647	Skunk 1,556/2,489	Gray fox * 106/282	Red fox 56/344	Woodchuck 96/1,424	Coyote 10/102	Cat 175/20,506	Dog 9/7,772	Cow 15/89
Aggression	664/305	597/180	79/34	30/36	41/186	6/5	74/6,638	5/4,482	2/2
OR †	**2.88**	**7.99**	**21.34**	**9.87**	**4.56**	**29.1**	**1.53**	0.92	6.90
p value	**<0.0001**	**<0.0001**	**<0.0001**	**<0.0001**	**<0.0001**	**<0.0001**	**0.0053**	0.90	0.099
Ataxia	134/186	40/69	5/33	2/34	4/32	0/2	69/2,394	4/409	5/37
OR	0.77	0.93	0.37	0.34	1.89	–	**4.92**	**14.4**	0.70
p value	0.026	0.76	0.053	0.20	0.28	–	**<0.0001**	**<0.0001**	0.54
Disorientation	542/582	302/273	29/101	8/96	24/112	2/14	70/3,186	3/545	6/34
OR	1.02	**1.95**	0.67	0.43	**3.90**	1.57	**3.62**	**5.68**	1.08
p value	0.81	**<0.0001**	0.12	0.031	**<0.0001**	0.59	**<0.0001**	**0.0045**	0.89
Found dead	253/232	218/560	7/30	2/43	5/232	2/15	7/676	0/125	1/5
OR	**1.21**	0.56	0.59	0.26	0.28	1.45	1.22	–	1.20
p value	**0.048**	<0.0001	0.23	0.065	0.33	0.66	0.52	–	0.87
Lethargy	243/407	76/157	1/49	3/66	16/62	0/14	51/3,044	3/403	5/24
OR	0.61	0.76	0.05	0.24	**4.39**	–	**2.36**	**7.84**	1.35
p value	<0.0001	0.061	<0.0001	0.011	**<0.0001**	–	**<0.0001**	**0.0004**	0.61
Paralysis	88/120	19/82	4/10	4/22	5/15	0/5	36/1,024	1/229	5/25
OR	0.79	0.36	1.07	1.13	**5.16**	–	**4.93**	3.66	1.28
p value	0.10	<0.0001	0.91	0.83	**0.0066**	–	**<0.0001**	0.26	0.68
Salivation	99/94	42/58	7/46	3/23	1/10	1/2	20/1,556	5/387	9/28
OR	1.15	1.16	0.36	0.79	1.49	5.56	1.57	**19.08**	**3.27**
p value	0.34	0.47	0.013	0.71	0.51	0.25	0.057	**<0.0001**	**0.033**
Seizure	57/77	19/60	1/60	1/32	1/7	1/1	10/1,646	1/507	0/11
OR	0.80	0.50	0.04	0.18	2.13	11.22	0.69	1.59	–
p value	0.22	0.0072	<0.0001	0.066	0.41	0.17	0.26	0.66	–

* 35 foxes not differentiable for species were excluded from the table, of which 3 were confirmed to be infected with RRV virus;

† Odds ratio of rabies for animals with reported signs vs. no reported signs;

§ Framed cell indicates significant association between rabies and observed signs.

**Table 2:** Predictive value of one or more reported clinical signs in the identification of RRV virus infection, by animal species, Massachusetts, 1992–2010.

Animal species (associated sign(s))	Diagnostic values			

	PPV * % (No.)	NPV ^#^ % (No.)	Accuracy ^&^ % (No.)	OR ^↑^ (95% CI)
Raccoon (aggression/found dead)	63.05% (906/1,437)	58.07% (2,116/3,644)	59.48% (3,022/5,081)	2.36 (2.08–2.68)
Skunk (aggression/disorientation)	64.38% (768/1,193)	72.37% (2,064/2,852)	70.01% (2,832/4,045)	4.73 (4.1–5.47)
Gray fox (aggression)	69.91% (79/113)	90.18% (248/275)	84.28% (327/388)	21.34 (12.13–37.56)
Red fox (aggression)	45.45% (30/66)	92.21% (308/334)	84.5% (338/400)	9.87 (5.23–18.51)
Woodchuck (aggression/	16.27%	96.94%	77.04%	6.16
disorientation/lethargy/paralysis)	(61/375)	(1,110/1,145)	(1,171/1,520)	(3.99–9.51)
Coyote (aggression)	54.55% (6/11)	96.04% (97/101)	91.96% (103/112)	29.1 (6.16–137.37)
Cat (ataxia/disorientation/lethargy/paralysis)	1.24% (142/11,476)	99.64% (9,172/9,205)	45.04% (9,314/20,681)	3.48 (2.38–5.09)
Dog (ataxia/disorientation/lethargy/salivation)	0.74% (7/945)	99.96% (6,834/6,837)	87.91% (6,841/7,782)	17 (4.39–65.85)
Cow (salivation)	24.32% (9/37)	91.04% (61/67)	67.31% (70/104)	3.27 (1.06–10.07)

* PPV (positive predictive value) = No. of rabid animals with any of the listed reported clinical signs/No. of total animals with any of the listed reported clinical signs;

# NPV (negative predictive value) = No. of non-rabid animals with none of the listed reported clinical signs/No. of total animals with none of the listed reported clinical signs;

& Accuracy = (No. of rabid animals with any of the listed reported clinical signs + No. of non-rabid animals with none of the reported clinical signs)/No. of testable animals;

† Odds ratio of rabies for animals with any versus none of the listed reported clinical signs.
